# Local Structural Distortion Induced Uniaxial Negative Thermal Expansion in Nanosized Semimetal Bismuth

**DOI:** 10.1002/advs.201600108

**Published:** 2016-06-01

**Authors:** Qiang Li, He Zhu, Lirong Zheng, Longlong Fan, Yang Ren, Jun Chen, Jinxia Deng, Xianran Xing

**Affiliations:** ^1^ Department of Physical ChemistryUniversity of Science and Technology BeijingBeijing100083China; ^2^ Beijing Synchrotron Radiation FacilityInstitute of High Energy PhysicsChinese Academy of SciencesBeijing100039China; ^3^ X‐Ray Science DivisionArgonne National LaboratoryArgonneIL60439USA

**Keywords:** EXAFS, local structural distortion, nanosized bismuth, negative thermal expansion, PDF

## Abstract

**The corrugated layer structure bismuth** has been successfully tailored into negative thermal expansion along *c* axis by size effect. Pair distribution function and extended X‐ray absorption fine structure are combined to reveal the local structural distortion for nanosized bismuth. The comprehensive method to identify the local structure of nanomaterials can benefit the regulating and controlling of thermal expansion in nanodivices.

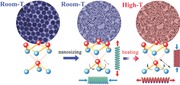

As one class of the most widely used materials, metal or semimetal occupies an important position in the field of study and application for engineering and functional materials. However, large thermal expansion of metal or semimetal sometimes causes a negative effect on the application in variable temperature environment.^1^ In order to maintain the thermal stability and physical properties, a universal and effective method to tailor the thermal expansion of metal or semimetal is strongly expected. So far, doping method to change the chemical composition is regarded as the most common way.^2^, ^3^, ^4^ The most famous example is invar alloy,^5^, ^6^ showing a low coefficient of thermal expansion (CTE) during magnetic transition. Furthermore, this kind of magnetic transition can also bring a negative thermal expansion (NTE) in an alloy such as La(Fe, Si)_13_‐based compounds.^7^

Unlike doping method, size effect recently has been proved to be another effective way to adjust the thermal expansion behaviors.^8^, ^9^, ^10^, ^11^ Because of the presence of dangling bonds, defects, and quantum correlation effects in nanosized metal or semimetal, some abnormal thermal expansion behaviors have been reported.^12^, ^13^ However, the large‐scale preparation of highly crystalline metal or semimetal powders within several nanometers and the controlling their CTEs are still challenging. Moreover, local structure study of these nanomaterials is essential, which is pivotal for better understanding of the NTE and related physical properties.

As a promising building block for next‐generation thermoelectric and spintronic devices, the nanosized bismuth and Bi‐based materials have been attracting extensive attention in the field of quantum transport, topological insulator and finite size effect.^14^, ^15^, ^16^ Similar to other group V elements, bismuth has the corrugated layer structure of rhombohedral A7 connected by so‐called metallic‐covalent bonds,^17^, ^18^ which is stabilized by Jones–Peielrs mechanism.^19^ Thermal expansion matching has always been the key issue to decide the effectiveness and cycling life in nanodevices, especially for the nanomaterials working under variable temperature like thermoelectric material. Despite such decisive role, the thermal expansion behaviors of nanosized bismuth has been paid little attention to. In bulk bismuth, positive thermal expansion along *a* and *c* axis can be found, defined as α_//_ and α_⊥_.^20^, ^21^ By contrast, a transition of CTE has been reported in bismuth nanowires, which was supposed to root in the discrete electronic energy levels.^22^ But for the lack of local structural distortion and electron structure information, the physical origin for such phenomenon is still unknown.

In this study, we investigated the thermal expansion of nanosized bismuth, which was synthesized by chemical reduction method.^23^, ^24^ Extended X‐ray absorption fine structure (EXAFS) and atom pair distribution function (PDF) analyses are incorporated to reveal the local structural distortions in nanosized bismuth. The relations among thermal expansion behaviors, local structural distortions, and chemical bonding were demonstrated and carefully discussed in virtue of ab initio calculations.

Bismuth nanoparticles with well crystalline were synthesized adopting oleylamine so as to reduce dodecyl mercaptan bismuth (see Figure S1 and Table S1, Supporting Information). The uniform morphology of as‐prepared bismuth particles is shown in the transmission electron microscope (TEM) images (see **Figure** **1**), with the average particles sizes of 8.9, 13.1, 28.5, and 111.7 nm, respectively. Due to the weak metallic‐covalent bonds in bismuth layer structure, 8.9 nm nanoparticles were of poor crystallinity from selected area electron diffraction (SAED) (see Figure 1c), which were excluded from following tests. The sub‐micrometer pie‐like bismuth particles of 112 nm were considered as the representative of bulk bismuth with Fermi wavelength of 30 nm^14^ (see Figure S2, Supporting Information). Rhombohedral A7 structure stabilized by Jones–Peielrs distortion^19^ leads to two atoms in a single primitive cell (see **Figure** **2**a). The X‐ray diffraction (XRD) patterns for bismuth nanoparticles and bulk ones indicate the same A7 structure (see Figure 2b), except for the broadening of peak shape from size effect in nanoparticles. The peak tail toward high angle around (012) peak can be identified as the disordered state from surface coating, which can almost be found in all bismuth nanoparticles synthesized by chemical method.^25^, ^26^

**Figure 1:**
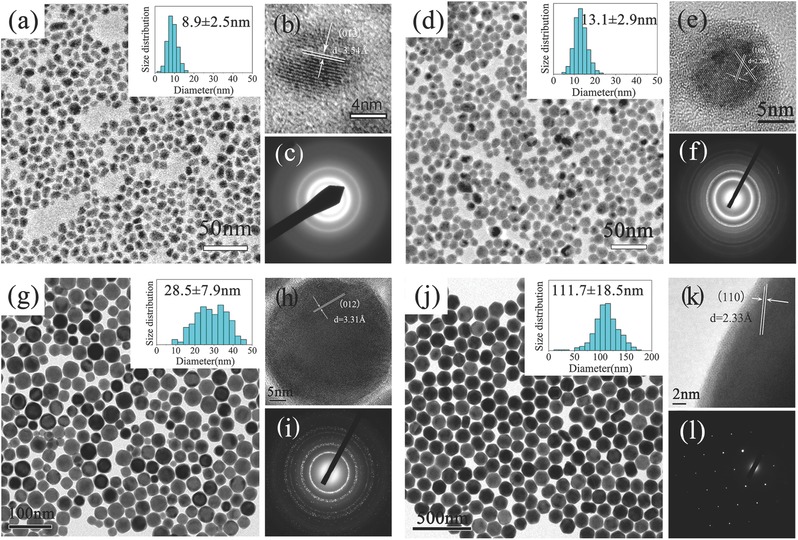
a–l) TEM, HRTEM, and SAED images of bismuth nanoparticles synthesized under different conditions. The details of synthesis conditions are listed in Table S1 of the Supporting Information. Insets of TEM images are diameter‐distribution histograms counting about 400 particles. The mean particle sizes are 8.9 ± 2.5, 13.1 ± 2.9, 28.5 ± 7.9, and 111.7 ± 18.5 nm, respectively.

**Figure 2:**
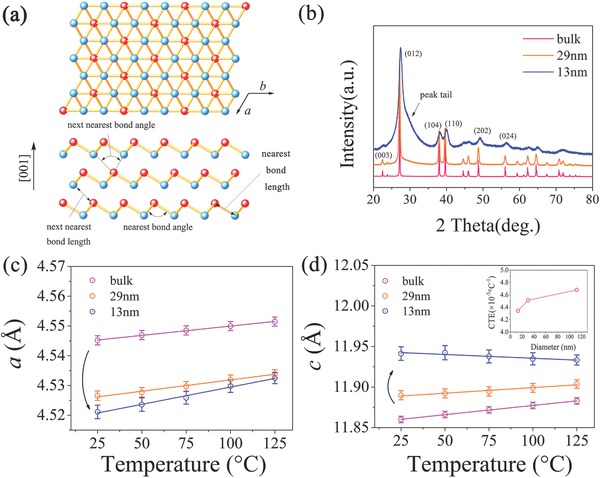
a) The layer structure for bulk bismuth of *R*‐3*m* space group. Two different colors represent the two atoms in a primitive cell. Yellow plain cylinders stand for metallic‐covalent bonds. (b) X‐ray diffraction patterns of the synthesized bismuth particles tested at room temperature. (c,d) Temperature dependences of lattice parameter *a* and *c* in hexagonal unit cell of bismuth particles. The size dependence of the coefficient of volume thermal expansion is shown in the inset of (d).

To avoid the growing of nanoparticles during heating, the maximum temperature for XRD measurements was selected as 125 °C according to the peak width. For the sake of extracting unit cell parameters and removing the effect of sample stage during heating, Lebail method^27^ were adopted to refine the XRD patterns, in which a general size model is included (see Table S2 and Figures S4–S6, Supporting Information). Despite many diffraction peaks of bismuth in the higher angle area, 80° was determined in the tests on account of the serious attenuation and broadening in high angle area for nanoparticles (see Figure S3, Supporting Information). At room temperature, *a* axis demonstrates a contraction while expansion for *c* axis with particle size decreases (see Figure 2c,d). The linear CTE along *c* axis was observed with a transparent decrease and a little increase for *a* axis. From bulk to 13 nm, coefficient of thermal expansion along *c* axis demonstrates a crossover from positive to negative, with the value of −0.784 × 10^−5^ °C^−1^. The opposite change tendency of α_//_ and α_⊥_ results in a slight decrease of the coefficient of volume thermal expansion (see the inset of Figure 2d).

In layer structure, a negative value of α_//_ is a common behavior, such as that in graphite and BN,^28^, ^29^, ^30^ ascribed to the bending acoustic waves with polarization vector perpendicular to the plane.^19^ Such collaborative effect named Poisson contraction is not conflicted with the expansion of chemical bonds. In our case, the negative thermal expansion was interesting to be found in the direction perpendicular to the layer plane of bismuth. Abnormal expansion behavior manifests different local structure in bismuth nanoparticles than that in bulk ones. In order to reveal the origin of this kind of unusual thermal expansion behaviors in nanosized bismuth, local structural information was extracted from EXAFS for its atomic selectivity and high sensitivity to the short‐range structure.^31^, ^32^, ^33^

The crumpled layer structure of bismuth can be simplified into a local structural unit consisting of nearest and next nearest bonding as the four scattering paths in EXAFS spectra (see inset of **Figure** **3**a). In order to isolate the pure size effect on local structure without thermal effect, the temperature of collecting EXAFS data is firstly chosen to be 10 K for the negligible effect of thermal vibration (Fourier transform of the experimental EXAFS patterns are showed in Figure 3a). Consistent with the results of XRD, nanosized bismuth particles have the bulk‐like local structure with four scattering paths above 3 Å, except for less intensity attributed to local structural disorder in the nanosized bismuth. With the decrease of particle size, an increasing abrupt coordination peak emerges before 3 Å, which represents the more chemical bonds on the surface of bismuth nanoparticle to stabilize its nanosize.^34^ Comparing the peak positions of four scattering paths in the Figure 3a, a small contraction of path 3 can be obtained while other paths almost keep the same. Fitting results (see Figure S7 and Table S3, Supporting Information) confirm a slight decrease of nearest and next nearest bond angles and the invariance of bond lengths when size decreases, indicating a more crumpled layer structure in the nanosized bismuth. Such change of the local structure precisely agrees with the change of unit cell with the decrease of particle size shown by XRD refinements (see Figure 3c). Although there are some differences for high coordination shells from short range disorder with size decreasing, the average local structure can be ensured as the same with bulk bismuth according to Raman spectra (see Figure 3b and Figure S9, Supporting Information). The peaks of 72 and 96 cm^−1^, called E_g_ mode and A_1g_ mode, represent the relative vibrations of two bismuth atoms in a primitive cell which are perpendicular and parallel to *c* axis, respectively. The softening for E_g_ mode and hardening for A_1g_ mode can be ascribed to the quantum confinement effects^35^ and change of bond strength. In order to survey the variation of coordination peaks with raising temperature, EXAFS tests of 100–300 K were also conducted (see Figure S8, Supporting Information). Different from the patterns of 10 K, coordination peaks of bismuth layer structure decay rapidly while only the first peak for surface bonding in nanosized particles remains unchanged. Similar situation can be also observed for 13 nm at 10 K (the fitting results are listed in Table S3, Supporting Information). Such attenuation due to static disorder from size effect and thermal disorder hinders the identification of coordination peaks from peak segments of Fourier transform.

**Figure 3:**
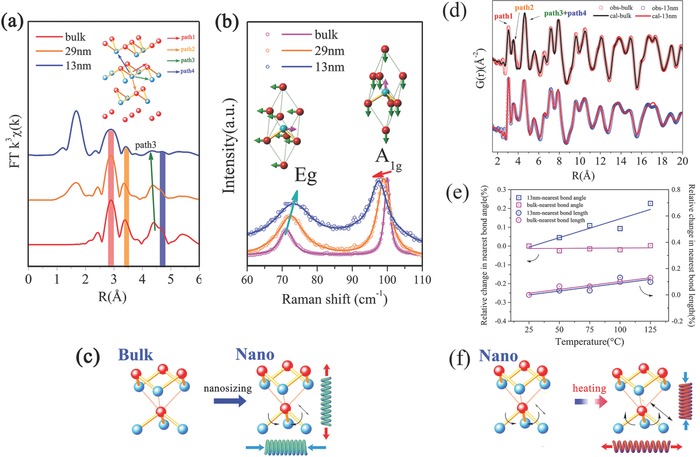
a) Fourier transforms of k^3^‐weighted Bi L_3_‐edge EXAFS spectra of bismuth particles at 10 K. The inset is the local structure of bismuth and the corresponding scattering paths in EXAFS. b) Raman spectra at 123 K and the vibration modes for bulk, 29 and 13 nm bismuth particles. c) The schematic diagram of the change of local structural distortions as particle size decreases. d) Pair distribution function of 13 nm bismuth nanoparticles and bulk particles at room temperature and the fitting results (*R*_w_ = 0.1233 and 0.0915, respectively). The paths correspond to the scattering paths showed in EXAFS. e) Temperature dependences of the relative change of nearest bond length and angle for 13 nm and bulk bismuth particles. f) The schematic diagram of the change of local structural distortions as heating for 13 nm particles.

Therefore, when comes into the field of nanoscience in which the traditional X‐ray fails for the limited coherence length, PDF method was incorporated. PDF could identify the interatomic correlations at much greater distances and have a stronger intensity and better signal‐to‐noise ratio than EXAFS.^36^, ^37^, ^38^, ^39^ The PDF data of 13 nm and bulk bismuth particles at room temperature are given in Figure 3d. Because of size effect, PDF for 13 nm particles displays the faster decay at high‐r part. Surface bonding is almost invisible in PDF for the weak scattering factor of organic surfactant and counteraction of 3D periodicity. In the short‐r side, local structural distortion from size effect, which is consistent with EXAFS, is retained (see Figure S11, Supporting Information). Comparing with the fit results of PDF for 13 nm and XRD for bulk bismuth, the different changing behaviors of local environment can be obtained (see Figure 3e and Figures S12 and S13, Supporting Information). Nearest bond lengths of nanosized and bulk bismuth both have the same dependence of temperature, except for a little offset. Due to the relation of first‐order derivative for CTE, thermal contribution of nearest bond length is little to the difference of thermal expansion along *c* axis. In contrast, nearest bond angle for 13 nm bismuth increases apparently while that almost keeps the same for bulk during heating. Such change of local structure introduces a contraction for intralayer in bismuth layer structure (see Figures S12 and S13, Supporting Information), which plays a decisive role in the thermal expansion along *c* axis (see Figure 3f).

Since there is no phase transformation from low temperature to high temperature, such local structural distortion revealed by EXAFS at low temperature can be extended to high temperature and finally induce the NTE of *c* axis. For further insight into the local structural distortion and uniaxial NTE for nanosized bismuth, ab initio calculations of the surfaces for bismuth were performed to reveal the role of chemical bonding and electron structure on the surface. The selected (001) and (100) surfaces, as the representatives of surface from natural cleavage and truncated surface, respectively, demonstrate transparent relaxation rather than reconstruction (see **Figure** **4**a and **Table** **1**).

**Figure 4:**
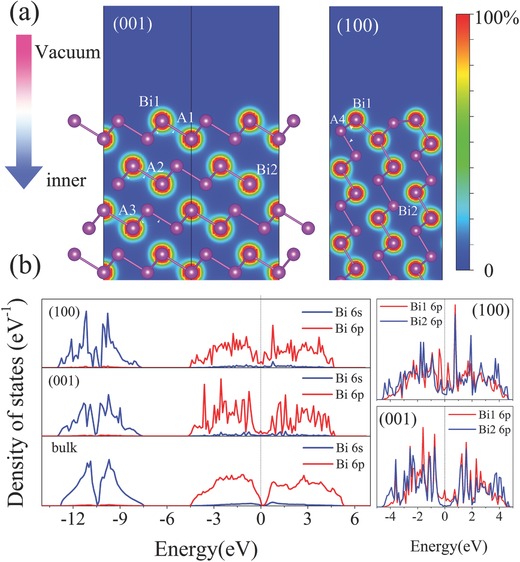
a) The calculated valence electron densities of the clean bismuth (001) and (100) surfaces. The 0% and 100% in color scale correspond to 0.02 and 1 eÅ^−3^. b) The calculated density of states for bulk and nanosized bismuth. The right part is the partial density of states for the surface atoms on the two bismuth surfaces.

**Table 1:** The bonding electron densities and corresponding local structure of the bismuth surfaces of (001) and (100)

	Bond length [Å]	Bond angle [°]	Bonding electron density [eÅ^−3^]
A1(001)	3.0827	95.0025	0.2897
A2(001)	3.0823	95.0133	0.2847
A3(001)	3.0783	95.1812	0.2842
A4(100)	3.0207	94.1039	0.3101

For (001) surface, the relaxation is indicated as a slight increase of the nearest bond length and a marked decrease of nearest bond angle, accompanied with the increase of the bonding electron densities (see Table 1). In addition, the stronger relaxation, attributed to more dangling bonds from truncated surface, can be pointed out for (100) surface. Similar relaxations can also be found in the other calculation results for the different surfaces.^40^, ^41^

For bismuth, the deep lying of 6s shell brings little contribution to metallic‐covalent bonds.^17^ The dominated 6p component determines the orientation of the bonds in bismuth as a crumpled layer structure.^42^ However, the more metallic character on the surfaces, as the higher surface carrier density around Fermi energy for two surfaces (see Figure 4b), demonstrates the more itinerant bonding electrons of 6p shell than that in inner part.^43^ The atomic partial density of states shows a transfer of 6p electrons from the bottom of band to Fermi energy for surface atoms like Bi1 than the inner Bi2, which identifies the main contribution of outermost atoms to metallic character. The itinerant character of bonding electrons means the weakening of covalent part for metallic‐covalent bonds on the surface and then reduces the repulsion and the orientation between the covalent bonds.^17^, ^42^, ^43^, ^44^ though the total electron density between the nearest atoms increases. This kind of change for electronic structure can be concluded as the reason of the structural distortion on the surface. For bismuth nanoparticles, surface becomes the dominated part. The local structural distortion on the surface contributes much more to the mean structure of bismuth than bulk. Moreover, the absorbate stabilizing nanoparticles with strong electronegativity on the surface (see Figure 3a and Figure S10, Supporting Information) can seize the bonding electrons from surface atoms resulting in weaker bonds on the surface and then aggravate the local structural distortion in nanosized bismuth. As thermal vibration intensifies during heating, such local structural distortion induces negative thermal expansion along *c* axis for nanosized bismuth.

In conclusion, the thermal expansion properties of semimetal bismuth have been successfully tailored by introducing size effect in the present study. A transition of the CTE from positive to negative can be observed along *c* axis in nanosized bismuth. The local structural distortions of the nanosized bismuth obtained from EXAFS and PDF reveal the key role of the nearest bonding environment, especially the nearest bond angle, which is responsible for the abnormal thermal expansion from other layer structure of strong bending acoustic waves with polarization vector perpendicular to the plane. Distortion as the decrease of nearest bond angle and increase of nearest bond angle from size effect then will relax under the drastic thermal activation with temperature rising. Further ab initio calculations for surfaces of different orientations showed the loss of covalent part for metallic‐covalent bonds on the surface, which induced the local structural distortion and resulted into the negative thermal expansion along *c* axis in nanosized bismuth. Our approach provides an example of controlling the thermal expansion behaviors of semimetal, which is of both scientific and technological importance for nanoscience.

## Experimental Section

*Synthesis*: Bi(NO_3_)_3_·5H_2_O(>99.9% in purity) was used as bismuth source during synthesis. Dodecyl mercaptan bismuth was synthesized by combining Bi(NO_3_)_3_·5H_2_O and 1‐dodecanethiol (Chemically Pure). Oleylamine (C18:80%–90%) was used as reductant and solvent. All synthetic steps were conducted under O_2_‐free Ar. The specific preparation conditions and quantities of reagents are listed in Table S1 of the Supporting Information. In a typical synthesis of 13 nm Bi nanoparticles, 0.5 g Bi(NO_3_)_3_·5H_2_O was added into 5 mL 1‐dodecanethiol to generate a yellow solution (there was some suspended solid in it possibly.). After stirred for 20 min in the water bath of 80 °C, the produced red transparent solution was injected 10 mL oleylamine. The solution turned quickly brown and then gradually darkened after the temperature of water bath was set as 70 °C. The final nanoparticles could be obtained after stirring in the water bath for 4 h. The schematic diagram of the experiment process is presented in Figure S1 of the Supporting Information.

*Measurements*: The as‐prepared Bismuth nanoparticles were isolated by adding acetone, followed by being centrifugated (9000 rpm, 3 min) and washed by acetone several times. The ultimate nanoparticles dispersed in methylbenzene were dropped on the carbon‐coated copper grid for morphology characterization of TEM (JEOL JEM‐2010 at 200kV) and SEM (scanning electron microscope, SUPRA‐40, Carl Zeiss). The crystal structures of particles with different sizes were confirmed by XRD (PW 3040‐X'Pert Pro, PANalytical, Cu Kα). Raman spectra were collected on LabRAM HR Evolution of HORIBA using laser with wavelength of 633 nm. Then for exacting the information of vibration peaks, fitting by two Lorentz peaks were conducted. As for characterizing the mean local structure distortion of nanoparticles, XAFS spectra were collected on the 1W1B beamline at Beijing Synchrotron Radiation Facility (BSRF). All samples were recorded at the Bi L_3_‐edge (E = 13419 eV) at 10, 100, 200, and 300 K with transmission mode. The later data processing and fitting were performed using the IFEFFIT program. The PDF analysis was obtained from high energy X‐ray scattering data by direct Fourier transform of reduced structure function with Q value of 18 Å^−1^ by program PDFGETX3, using the 11‐ID‐C beamline at the Advanced Photon Source (APS) of Argonne National Laboratory with the wavelength of 0.11165 Å. In addition, the surface composition and chemical state analysis were conducted with XPS (shown in Figure S10, Supporting Information, X‐ray photoelectron spectroscopy, AXIS ULTRADLD, Al Kα).

*Calculation Method*: For ab initio calculations of clean bismuth (001) and (100) surface with a vacuum layer of 15 Å we used the Perdew–Burke–Ernzerh of exchange–correlation functional within the generalized gradient approximation (GGA) and the projector‐augmented waves (PAW) method as implemented in Vienna ab initio Simulation Package (VASP) .The cut‐off energy for the basis set was 250 eV. The Brillouin zone was sampled by a 3 × 3 × 1 k‐mesh. The evaluation of the surface relaxation has been carried out for the film of 8 crumpled layer structure.

## Supporting information

As a service to our authors and readers, this journal provides supporting information supplied by the authors. Such materials are peer reviewed and may be re‐organized for online delivery, but are not copy‐edited or typeset. Technical support issues arising from supporting information (other than missing files) should be addressed to the authors.

Supplementary
