# Supplementary material for: Local Structural Distortion Induced Uniaxial Negative Thermal Expansion in Nanosized Semimetal Bismuth
Source: Adv Sci (Weinh). 2016 Jun 1;3(11):1600108. doi: 10.1002/advs.201600108 (PMC5102662; doi:10.1002/advs.201600108)
Supplement: Supplementary file 1 — Supplementary [file ADVS-3-0k-s001.pdf]

Copyright WILEY-VCH Verlag GmbH & Co. KGaA, 69469 Weinheim, Germany, 2016.

ADVANCED SCIENCE Open Access

Supporting Information

for *Adv. Sci.,* DOI: 10.1002/advs.201600108

Local Structural Distortion Induced Uniaxial Negative Thermal Expansion in Nanosized Semimetal Bismuth

*Qiang Li, He Zhu, Lirong Zheng, Longlong Fan, Yang Ren, Jun Chen, Jinxia Deng, and Xianran Xing**

Supporting Information

Local structural distortion induced uniaxial negative thermal expansion in nanosized semimetal bismuth

Qiang Li[†], *He Zhu*[†], *Lirong Zheng*[‡], *Longlong Fan*[†], *Yang Ren*[§], *Jun Chen*[†], *Jinxia Deng*[†], *Xianran Xing*[†,*]

†Department of Physical Chemistry, University of Science and Technology Beijing, Beijing 100083, China

‡Beijing Synchrotron Radiation Facility, Institute of High Energy Physics, Chinese Academy of Sciences, Beijing, 100049, China

§X-Ray Science Division, Argonne National Laboratory, Argonne, Illinois 60439, United States.

Email: xing@ustb.edu.cn

1. Experimental section

Table S1. The detailed synthesis conditions of bismuth nanoparticles and bulk particles

$Bi(NO_3)_3·5H_2O$(g)	1-dodecanethiol (ml)	Oleylamine (ml)	Reaction temperature(°C)	Reaction time(h)	Mean diameter(nm)
0.5	5	10	60	4	8.9±2.5
0.5	5	10	70	4	13.1±2.9
0.5	5	10	90	2	28.5±7.9
0.1	10	2.5	80	0.5	111.7±18.5

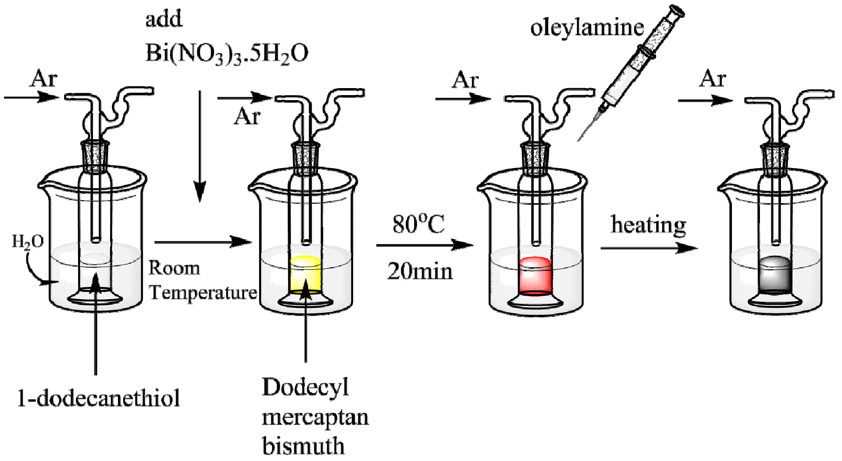

Figure S1. Schematic diagram of synthetic route for bismuth nanoparticles.

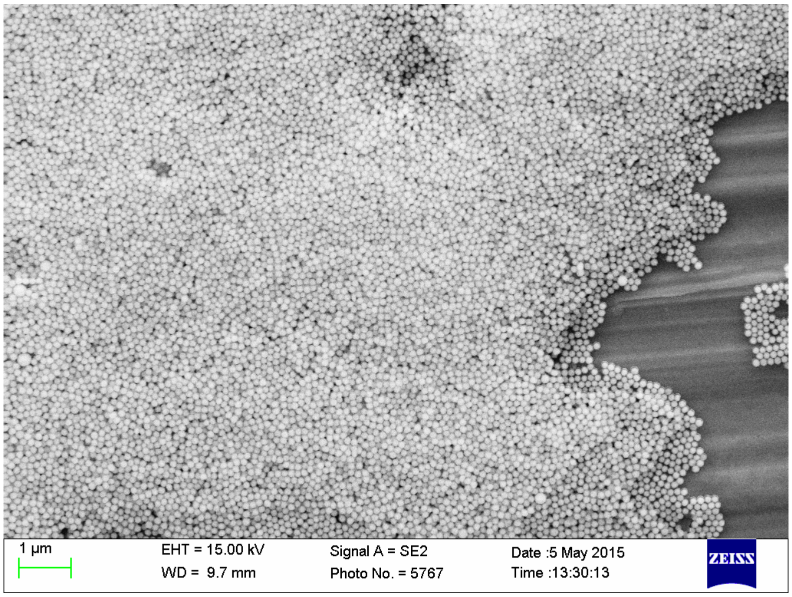

Figure S2. SEM image of 112 nm bismuth particles of low magnification.

2. Results and discussion.

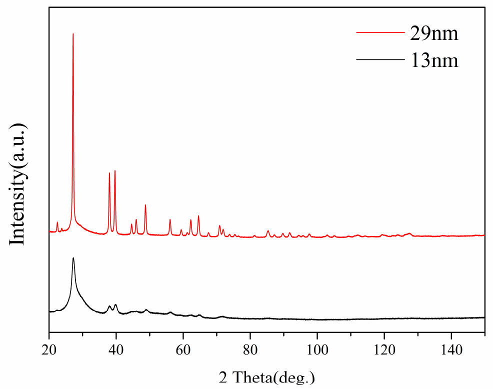

Figure S3. X-ray diffraction patterns of 13 nm and 29 nm bismuth particles with a wide range of angle at room temperature.

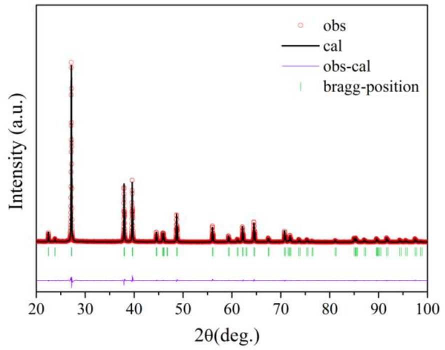

Figure S4. The observed (circles), calculated (line) and differential (bottom of figure) X-ray powder diffraction profiles of bulk bismuth particles from Rietveld refinement (Rp=8.82%, Rwp=7.99%).

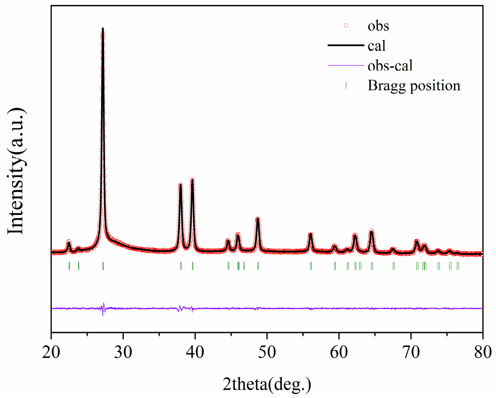

Figure S5. The observed (circles), calculated (line) and differential (bottom of figure) X-ray powder diffraction profiles of bismuth nanoparticles of 29 nm from Lebail refinement (Rp=9.71%, Rwp=9.36%).

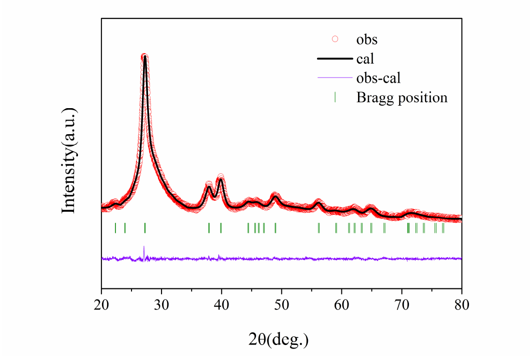

Figure S6. The observed (circles), calculated (line) and differential (bottom of figure) x-ray powder diffraction profiles of bismuth nanoparticles of 13 nm from Lebail refinement (Rp=8.36%, Rwp=8.31%).

Table S2. The refinement results of bismuth particles.

	bulk				29nm			13nm		
Temperature(°C)	a	c	z	Rwp	a	c	Rwp	a	c	Rwp
25	4.54566(1)	11.86146(4)	0.23382(1)	7.99	4.52656(8)	11.8898(3)	9.36	4.5211(2)	11.9412(7)	8.31
50	4.54693(1)	11.86608(4)	0.23386(1)	8.01	4.52771(8)	11.8921(3)	9.21	4.5235(2)	11.9423(8)	8.58
75	4.54848(1)	11.87185(4)	0.23383(1)	8.18	4.52971(8)	11.8946(3)	9.42	4.5255(2)	11.9377(7)	8.22
100	4.54996(1)	11.87734(4)	0.23383(1)	7.90	4.53193(8)	11.8989(3)	9.23	4.5297(2)	11.9348(7)	8.33
125	4.55146(1)	11.88283(4)	0.23378(1)	7.92	4.53391(7)	11.9036(3)	10.3	4.5324(1)	11.9333(6)	8.08

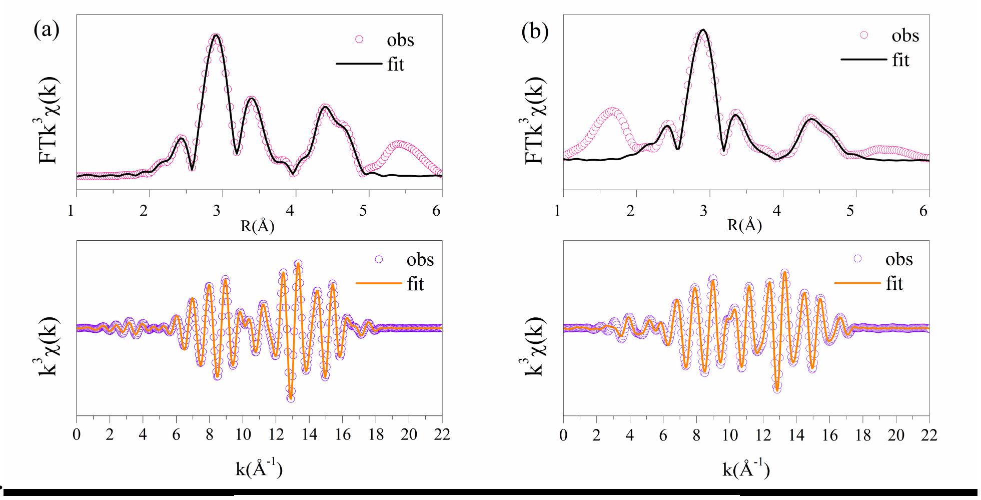

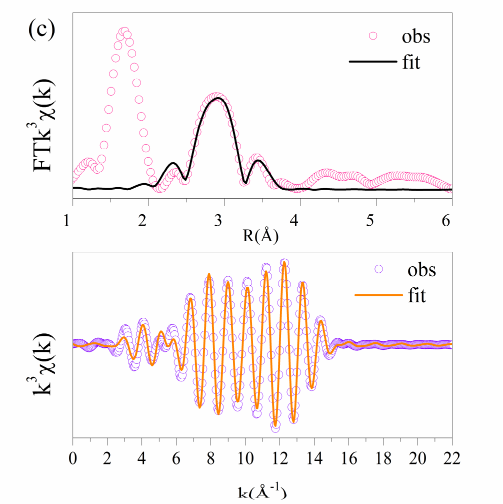

Figure S7. (a-c) The fitting results for Fourier transforms of k^3-weighted Bi L_3-edge EXAFS spectra of the bulk, 29nm and 13nm nanoparticles at 10 K.

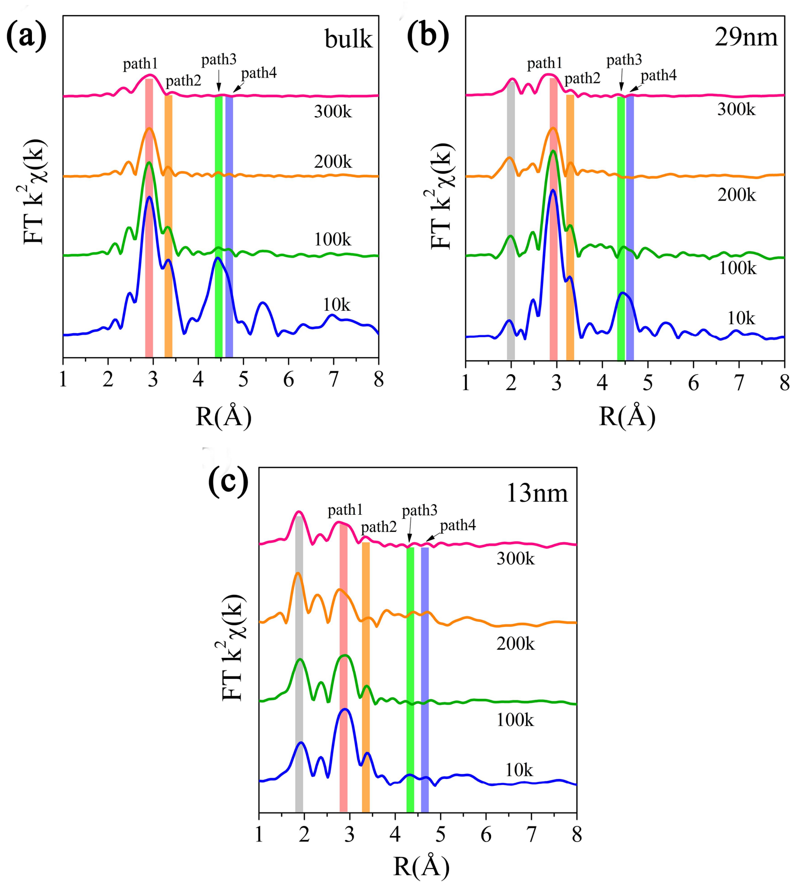

Figure S8. Fourier transforms of k^2-weighted Bi L_3-edge EXAFS spectra of the bismuth particles at 10 K, 100K, 200K and 300K. The tags correspond to the scattering paths with the same colors. The gray tag before the nearest coordination shows the surface bonding as mentioned in article.

Table S3. The structural parameters at 10 k for the different scattering paths extracted from the fits of EXAFS and the path1 parameter for bismuth nanoparticles. The lattice parameters listed in the table were calculated based on the scattering path parameters.

	Bulk(10k)	σ^2(Å^2)	29nm(10k)	σ^2
Path1(Å)	3.059	0.0016	3.060	0.0018
Path2(Å)	3.510	0.0027	3.509	0.0059
Path3(Å)	4.531	0.0032	4.523	0.0048
Path4(Å)	4.727	0.0032	4.724	0.0061
R-factor	0.00445		0.00985	
a(Å)	4.531		4.523	
c(Å)	11.778		11.815	
z	0.2340		0.2342	
Nearest bond length(Å)	3.059		3.060	
Nearest bond angle(°)	95.571		95.305	
Next nearest bond length(Å)	3.510		3.508	

Next nearest bond angle(°)	80.404			80.28		
	Path1-bulk		Path1-29nm		Path1-13nm	
Temperature(K)	bondlength(Å)	σ^2(Å^2)	bondlength(Å)	σ^2(Å^2)	bondlength(Å)	σ^2(Å^2)
10K	3.059	0.0016	3.060	0.0018	3.067	0.0045
100K	3.061	0.0029	3.060	0.0030	3.124	0.0091
200K	3.058	0.0050	3.053	0.0049	3.071	0.0077
300K	3.076	0.0072	3.071	0.0073		

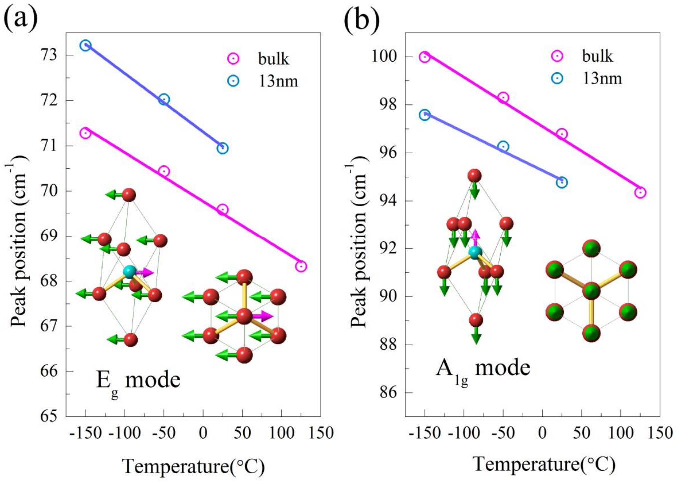

Figure S9. The temperature dependence of the two vibration modes for the particles of bulk and 13 nm.(a) E_g mode, (b) A_{1g} mode.

Because of the weak metallic-covalent bonds in bismuth, the increase of temperature brings a serious attenuation of Raman peak intensity, especially for 13nm. So the testing for nanosized bismuth was just conducted from -150℃to 50℃. From the figure above, due to the change of nearest bond angle and length in nanosized bismuth, the softening of E_g mode as vibration perpendicular to *c* axis will become more rapid while slower for A_{1g}.

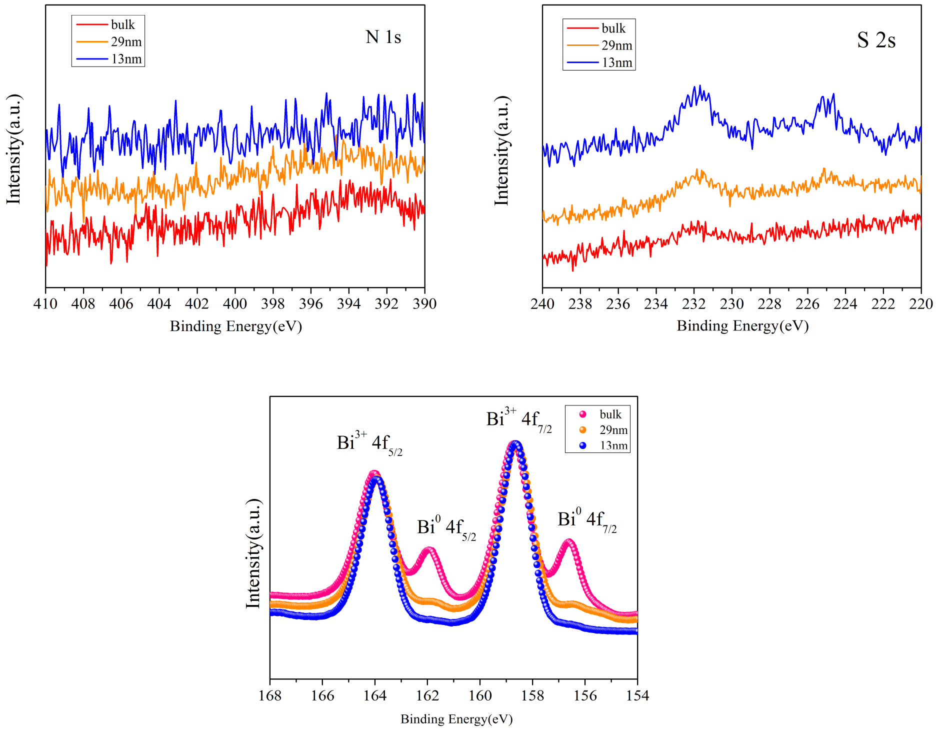

Figure S10. XPS spectra of the as-prepared particles showing the N 1s, S 2s and Bi 4f peaks.

XPS spectra suggest no obvious peaks of N 1s, meaning no oleylamine coating on the surface of nanoparticles. On the other hand, more apparent peaks of S 2s can be observed in the XPS spectrum which indicates the presence of 1-dodecanethiol on the surface. The peak on the side of low binding energy represents the thiolates while sulfonates on the high energy side. Because of the existence of S atoms and O atoms on the surface, the peak intensities for trivalent bismuth gradually increase with the decrease of particle size.

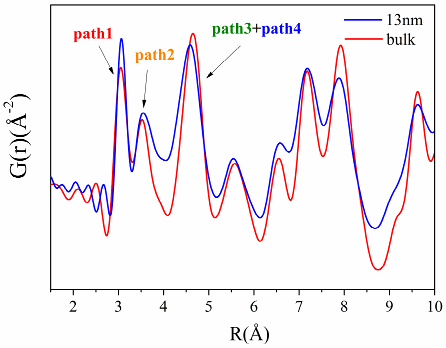

Figure S11. The short-r part of pair distribution function for bulk and 13nm bismuth particles.

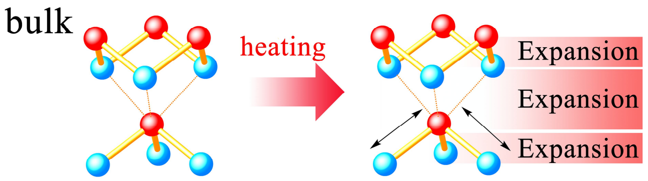

Figure S12. Schematic diagram of the change of local structural distortions as heating for bulk bismuth.

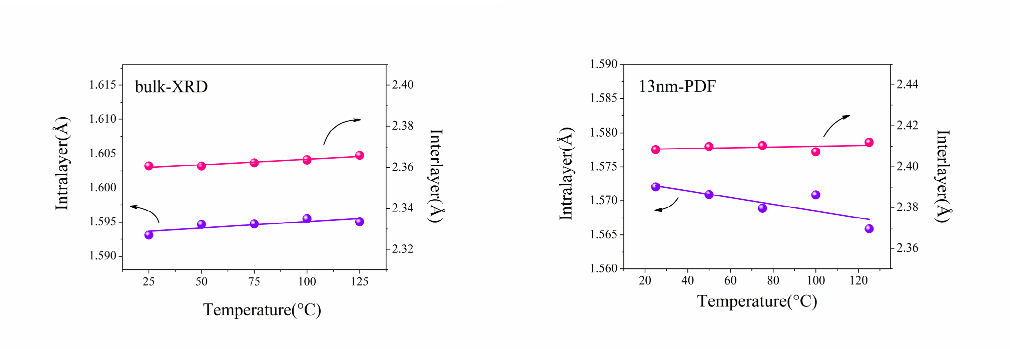

Figure S13. The temperature dependences of intralayer and interlayer spaces for bulk (XRD) and 13nm nanosized bismuth (PDF).

.

Except for the different behaviors of nearest structure which contribute to the thermal expansion, the next nearest structure of the particles shows the similar performance which can be regarded as insignificant parts to the thermal expansion.
